# Genome of Pseudomonas sp. FeS53a, a Putative Plant Growth-Promoting Bacterium Associated with Rice Grown in Iron-Stressed Soils

**DOI:** 10.1128/genomeA.00248-15

**Published:** 2015-04-02

**Authors:** Rocheli de Souza, Fernando Hayashi Sant’Anna, Adriana Ambrosini, Michele Tadra-Sfeir, Helisson Faoro, Fabio Oliveira Pedrosa, Emanuel Maltempi Souza, Luciane M. P. Passaglia

**Affiliations:** a Departamento de Genética, Instituto de Biociências, Universidade Federal do Rio Grande do Sul (UFRGS), Porto Alegre, RS, Brazil; b Departamento de Bioquímica e Biologia Molecular, Universidade Federal do Paraná (UFPR), Centro Politécnico, Curitiba, PR, Brazil

## Abstract

Pseudomonas sp. FeS53a was isolated from the roots of rice plants cultivated in one area with a well-established history of iron toxicity. The FeS53a genome sequence provides the genetic basis for understanding its lifestyle and survival in association with rice in conditions of iron toxicity.

## GENOME ANNOUNCEMENT

Plant-microbe interactions in the rhizosphere are the determinants of plant health and productivity and soil fertility (1–3). Plant growth-promoting bacteria promote plant growth, as well as provide bioprotection against biotic and abiotic stresses. Several studies have been conducted addressing the positive effects of bacterial strains in plants grown under abiotic stress (4–8), particularly with the Pseudomonas genus (5, 9–16).

Pseudomonas sp. FeS53a was isolated from the roots of rice plants collected in Camaquã (30°54′07.96″S, 51°51′26.25″W), which is an area with a well-established history of iron toxicity in the state of Rio Grande do Sul, Brazil (8). Approximately 329 diverse bacterial strains (26 bacterial genera) were selectively isolated based on their growth in selective media to investigate their genetic and functional diversity. The Pseudomonas genus was one of the most prominent bacterial isolates, and it possesses plant growth-promoting abilities, such as siderophore and indolic compound production and phosphate solubilization (8).

The genomic DNA of strain FeS53a was extracted and constructed into a 500- to 1,200-bp insert library. The whole-genome sequencing was performed with MiSeq Illumina platforms using the MiSeq reagent kit v2. The assembly was initially tested using 4 software packages—CLC Genomics Workbench, A5-miseq (17), CISA (18), and SPAdes (19); the A5-miseq was chosen due to the lower value of *N*_50_, and the small number of contigs. To assess the quality of the microbial genome, the CheckM program (20) was used with a completeness of 99.35%. Finally, the automatic annotation and classification of sequences were obtained using the RAST Server (21).

The sequencing of FeS53a resulted in 956,874 high-quality paired-end reads (approximately 59-fold coverage). The 95 contigs obtained were connected to form 85 scaffolds based on the paired-end relationships of the reads. The draft genome sequence of strain FeS53a is composed of 5,937,411 bp, with a G+C content of approximately 67.29%. Fifty-six tRNA genes, 7 rRNA genes, and 5,404 coding sequences (CDSs) were assigned on the basis of the annotation.

The FeS53a genome contains genes involved in the route of auxin biosynthesis, and these genes can sustain metabolic processes and aid in plant growth promotion under abiotic stress conditions. This strain encodes antioxidant enzymes, such as superoxide dismutase and catalase, which have the ability to remove free radicals and to prevent damage to the membranes and DNA, acquiring importance in abiotic stress management. The genome contains genes involved in iron storage systems (bacterioferritin), and there is also a ferric uptake regulation protein (Fur). The genome sequencing reported in this study will facilitate a comprehensive understanding of the lifestyle and survival of this strain and the plant-microbe interaction mechanisms under conditions of iron stress.

### Nucleotide sequence accession numbers.

This whole-genome shotgun project has been deposited at GenBank under the accession number JYFT00000000. The version described in this paper is version JYFT01000000.
